# DiCleave: a deep learning model for predicting human Dicer cleavage sites

**DOI:** 10.1186/s12859-024-05638-4

**Published:** 2024-01-09

**Authors:** Lixuan Mu, Jiangning Song, Tatsuya Akutsu, Tomoya Mori

**Affiliations:** 1 Bioinformatics Center, Institute for Chemical Research, Kyoto University, Kyoto, 611-0011 Japan; 2 Monash Biomedicine Discovery Institute and Department of Biochemistry and Molecular Biology, Monash University, Melbourne, VIC 3800 Australia

**Keywords:** miRNA, Dicer cleavage site prediction, Deep learning, Autoencoder

## Abstract

**Background:**

MicroRNAs (miRNAs) are a class of non-coding RNAs that play a pivotal role as gene expression regulators. These miRNAs are typically approximately 20 to 25 nucleotides long. The maturation of miRNAs requires Dicer cleavage at specific sites within the precursor miRNAs (pre-miRNAs). Recent advances in machine learning-based approaches for cleavage site prediction, such as PHDcleav and LBSizeCleav, have been reported. ReCGBM, a gradient boosting-based model, demonstrates superior performance compared with existing methods. Nonetheless, ReCGBM operates solely as a binary classifier despite the presence of two cleavage sites in a typical pre-miRNA. Previous approaches have focused on utilizing only a fraction of the structural information in pre-miRNAs, often overlooking comprehensive secondary structure information. There is a compelling need for the development of a novel model to address these limitations.

**Results:**

In this study, we developed a deep learning model for predicting the presence of a Dicer cleavage site within a pre-miRNA segment. This model was enhanced by an autoencoder that learned the secondary structure embeddings of pre-miRNA. Benchmarking experiments demonstrated that the performance of our model was comparable to that of ReCGBM in the binary classification tasks. In addition, our model excelled in multi-class classification tasks, making it a more versatile and practical solution than ReCGBM.

**Conclusions:**

Our proposed model exhibited superior performance compared with the current state-of-the-art model, underscoring the effectiveness of a deep learning approach in predicting Dicer cleavage sites. Furthermore, our model could be trained using only sequence and secondary structure information. Its capacity to accommodate multi-class classification tasks has enhanced the practical utility of our model.

**Supplementary Information:**

The online version contains supplementary material available at 10.1186/s12859-024-05638-4.

## Background

MicroRNAs (miRNAs) are a class of non-coding RNAs that function as gene expression regulators across various animal model systems [1]. Typically, miRNAs interact with the 3’ untranslated region of target mRNAs, leading to mRNA degradation and the downregulation of gene expression [2]. The dysregulated miRNA expression is closely associated with a spectrum of diseases including cancer [3–5], cardiovascular diseases [6, 7], inflammatory diseases [8–10], neurodevelopmental disorders [11–13], and others [14].

Mature miRNAs are approximately 20 to 25 nucleotides long. In the canonical biogenetic pathway of mature miRNAs, genes related to miRNAs are transcribed into much longer RNAs known as primary miRNAs (pri-miRNAs), which can extend up to several kilobases in length [15]. These pri-miRNAs undergo processing by a microprocessor complex comprising Drosha, a ribonuclease (RNase) III enzyme, and its associated helper proteins [16]. This processing step results in the generation of short RNAs termed precursor RNAs (pre-miRNAs). Subsequently, an RNase III endonuclease called Dicer cleaves the pre-miRNAs, giving rise to mature miRNAs [1]. The cleavage of pre-miRNAs produces two mature miRNA strands, one originating from the 5’ arm and the other from the 3’ arm of the pre-miRNAs [17].

Several machine learning-based approaches that primarily focus on protein cleavage sites have emerged for cleavage site prediction [18–27]. Two support vector machine (SVM) models were introduced for human Dicer cleavage site prediction [28, 29]. Liu et al. proposed a gradient boosting machine model called ReCGBM [30], which predicts the presence of a cleavage site within the central portion of a 14-nucleotide (nt) sequence. Although ReCGBM surpasses other SVM-based models in performance, there is still room for further enhancement. Firstly, ReCGBM operates solely as a binary classifier, which means it can determine whether a given segment contains a cleavage site but cannot discern the originating arm of the segment. This limitation is significant because pre-miRNAs contain two Dicer cleavage sites, thereby constraining the applicability of ReCGBM within an intricate end-to-end system. Secondly, ReCGBM underutilizes structural information inherent in pre-miRNAs although the secondary structure plays an important role in RNA function [31, 32].

In recent machine learning research, deep neural networks (DNNs) have demonstrated proficiency in computer vision and natural language processing, successfully finding applications in various bioinformatics studies. Their capacity to extract latent features from input data has rendered them particularly valuable. One notable advantage of employing computational models lies in the ability of the models to offer a practical and dependable alternative to laborious and expensive experiments, such as RNA structure prediction [33, 34] and drug-drug interaction prediction [35, 36]. Furthermore, extensive investigations have been conducted on the application of deep learning models to bioinformatics and computational biology [37–39].

In response to the limitations observed in ReCGBM, we introduce DiCleave, a DNN-based model. DiCleave combines pre-miRNA sequence segments with their secondary structure information facilitated by an autoencoder. Our model operates as both a binary classifier and a multi-class classifier, and its performance is comparable to that of ReCGBM in binary classification tasks. Our proposed multi-class classification model also demonstrates compelling performance.

## Methods

### Concept of cleavage pattern

**Fig. 1:**
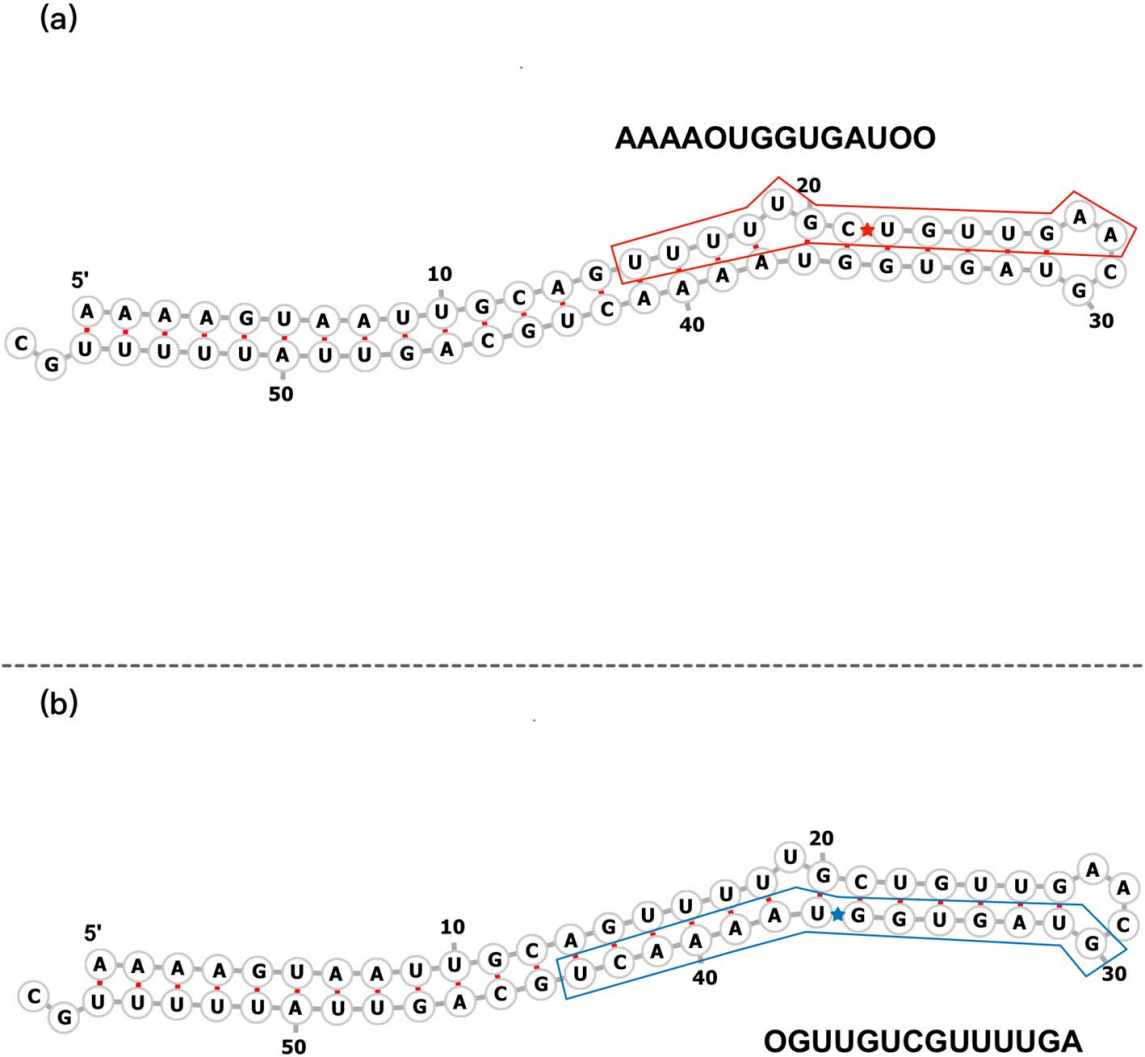
Illustrations depicting a cleavage window and its corresponding complementary sequence are presented. The predicted secondary structure of hsa-mir-548ar is shown as an example. **a** The 5’ end cleavage window is denoted by the red region, consisting of a 14-nt sequence. The Dicer cleavage site is indicated by a red asterisk positioned at the center of this region. The complementary sequence of the 5’ end cleavage window is shown above. It is worth noting that the fifth and the last two bases (U, A, A, respectively) remain unpaired. To represent this structural feature, we employ the addition symbol (O). **b** The 3’ end cleavage window is highlighted in blue, and the 3’ Dicer cleavage site is denoted by a blue asterisk positioned at its center. The complementary sequence of the 3’ end cleavage window is shown below. The unpaired first guanine (G) in this sequence is represented by the symbol O

ReCGBM employs a 14-nt-long segment of pre-miRNA as an input, which we refer to as the “cleavage pattern”. A cleavage pattern is categorized as positive if it has a Dicer cleavage site at its center. Conversely, it is designated as negative in the absence of a Dicer cleavage site. Pre-miRNA naturally folds into its secondary structure, resulting in the existence of a “complementary sequence” corresponding to each cleavage pattern. If a base within a cleavage pattern is paired with another base, the corresponding base is part of the complementary sequence and denoted by its standard nucleotide symbol (“A”, “C”, “G”, or “U”). Conversely, if no pairing occurs, the corresponding base in the complementary sequence is designated as “O”. An illustrative example of a cleavage pattern and its corresponding complementary sequence is presented in Fig. 1.

### Main dataset

We utilized a total of 956 human pre-miRNA sequences sourced from ReCGBM [30], which were obtained from the miRBase database (Release 22.1) [40], to form the main dataset in this study. Each pre-miRNA sequence had two positive cleavage patterns: one derived from the 5’ arm and the other from the 3’ arm. We randomly selected two 14-nt sequences from each pre-miRNA that did not contain the Dicer cleavage site, one from the 5’ arm and the other from the 3’ arm, and designated them as negative cleavage patterns. As a result, each pre-miRNA yielded four distinct cleavage pattern entities, resulting in a cumulative collection of 3,824 entities for our study. Furthermore, we accessed miRBase (Release 22.1, the latest release at the time) to obtain the full-length sequences of these human pre-miRNAs. The full-length pre-miRNA sequences were subsequently processed using RNAfold, an RNA secondary structure prediction tool available from ViennaRNA [41], to derive the secondary structures of the pre-miRNAs in the dot-bracket format. The configuration of the main dataset is presented in Table 1.

**Table 1:** Configuration of the main dataset

Column Header	Description	Example
Pat	14-nt-long cleavage pattern sequence	AGUGAUUGUCUUCC
ID	Pre-miRNA ID in miRBase	hsa-mir-4704
Seq	Full-length pre-miRNA sequence	CUUAUCCUAGACACUAGGCAUGUGAGUGAUUGUCUUCCUCACUCAAUCAGUCACAUAUCUAGUGUCUAGAAUGAG
Dot-Seq	Pre-miRNA secondary structure	(((((.((((((((((((.((((((.((((((………..)))))).)))))).)))))))))))).)))))
Dot-Pat	Secondary structure of cleavage pattern	(.((((((……
Comp	Complementary sequence	UOACUAACOOOOOO
Arm	In which arm is the cleavage pattern located?	5
Label*	Whether containing a cleavage site or not	1

^*^In the binary classification task, the patterns containing a cleavage site are labeled 1, whereas those lacking a cleavage site are labeled 0. In the multi-class classification task, the positive patterns from the 5’ arm are labeled 1, whereas those from the 3' arm are labeled 2

### Autoencoder dataset

We trained an autoencoder to extract the secondary structure embeddings of pre-miRNAs. We retrieved all pre-miRNA sequences from miRBase and excluded those already present in the main dataset. Subsequently, we filtered the remaining sequences by their lengths, retaining those that are 40 to 200 nucleotides long. These selected sequences were then subjected to RNAfold to generate their corresponding secondary structures. This process resulted in the compilation of 36,190 pre-miRNA secondary structures for the autoencoder training.

### Data preprocessing

RNAfold generates predicted secondary structures in the dot-bracket format employing three symbols: the left bracket, “(”, the right bracket,”)”, and the dot “.”, where pairs of left and right brackets indicate matching base pairs, and dots represent unpaired bases. To represent the secondary structures, we utilized one-hot encoding. The autoencoder received a fixed 200-nt-long sequence as the input, whereby any sequence shorter than 200 nt was supplemented with the placeholder “N”. Consequently, pre-miRNA secondary structures were encoded using four symbols: “(”, “)”, “.”, and “N”, yielding a 4 × 200 tensor (Additional file 1**: Fig. S1(a)**).

We similarly employed one-hot encoding to represent the cleavage pattern sequences. As illustrated in Fig. 1, when a base in the pattern sequence remained unpaired, the corresponding position in the complementary sequence was denoted by the symbol “O”. Consequently, both the cleavage patterns and their complementary sequences were encoded using five symbols: “A”, “C”, “G”, “U”, and “O”. The dot-bracket notation of the secondary structure of the cleavage pattern was one-hot encoded with three symbols without the use of the placeholder “N”.

### Datasets separation

To facilitate the training and evaluation of the binary classification models, we divided the main dataset into two distinct sub-datasets. One sub-dataset comprised all patterns originating from the 5’ arm whereas the other contained patterns from the 3’ arm. From the 5’ arm sub-dataset, we randomly selected 800 positive and 800 negative patterns to form a training dataset referred to as 5p_train. The remaining 312 patterns were reserved for an independent test set designated as 5p_test. A similar process was applied to the patterns from the 3’ arm, resulting in the creation of 3p_train and 3p_test sets.

**Fig. 2:**
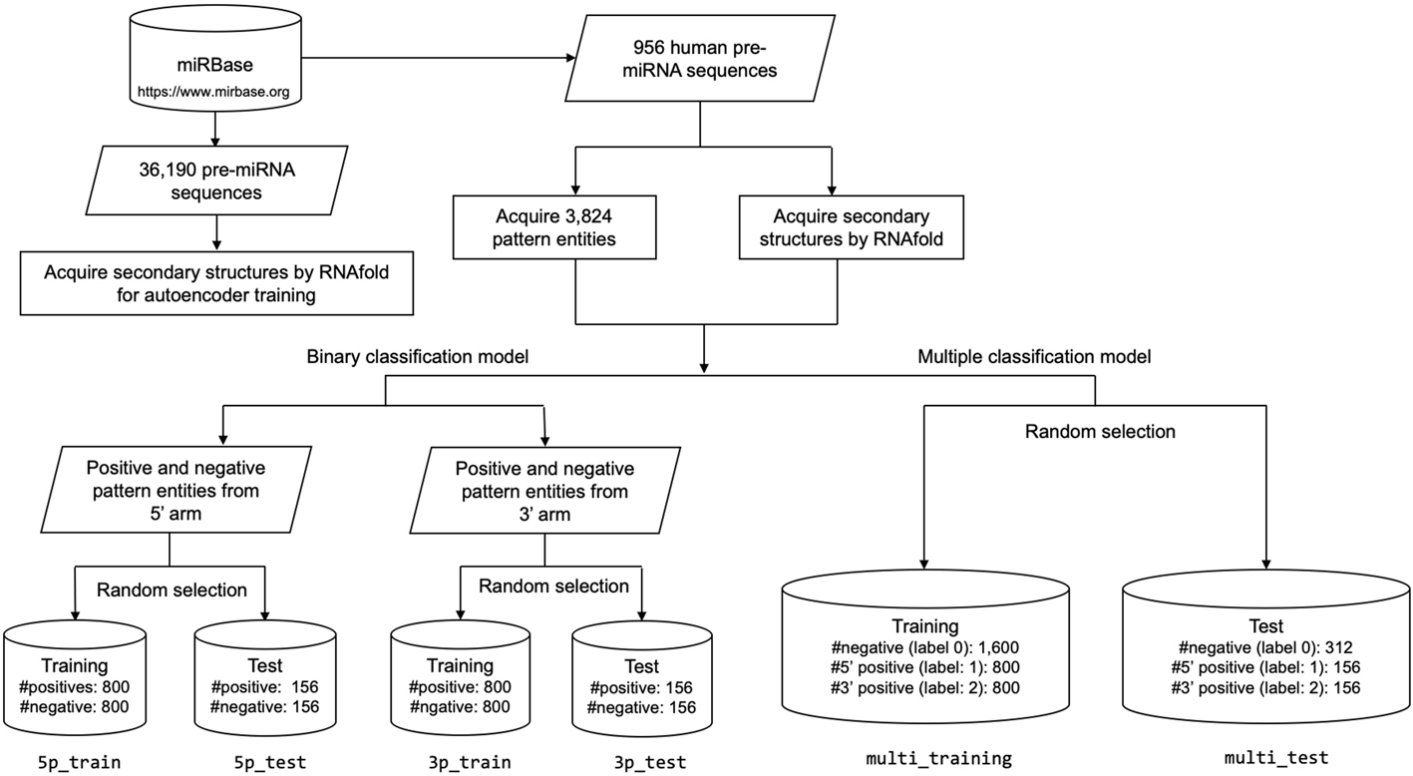
An overview of data processing and partitioning for the binary and multi-class classification tasks

To construct sub-datasets for the multi-class classification model, we redefined the labels of the patterns in the main dataset. The negative patterns and the 5’ positive patterns retained labels 0 and 1, respectively, whereas the labels of the 3’ positive patterns were modified to 2. Subsequently, we randomly selected 800 patterns each from the 5’ positive set and the 3’ positive sets and 800 negative patterns each from the 5’ arm and the 3’ arm. As a result, a total of 3,200 patterns were designated as the training set (multi-training), whereas the remaining 624 patterns were set aside as an independent test set (multi-test) for multi-class classification. An overview of the data processing and partitioning is shown in Fig. 2**.**

### Architecture of autoencoder

**Fig. 3:**
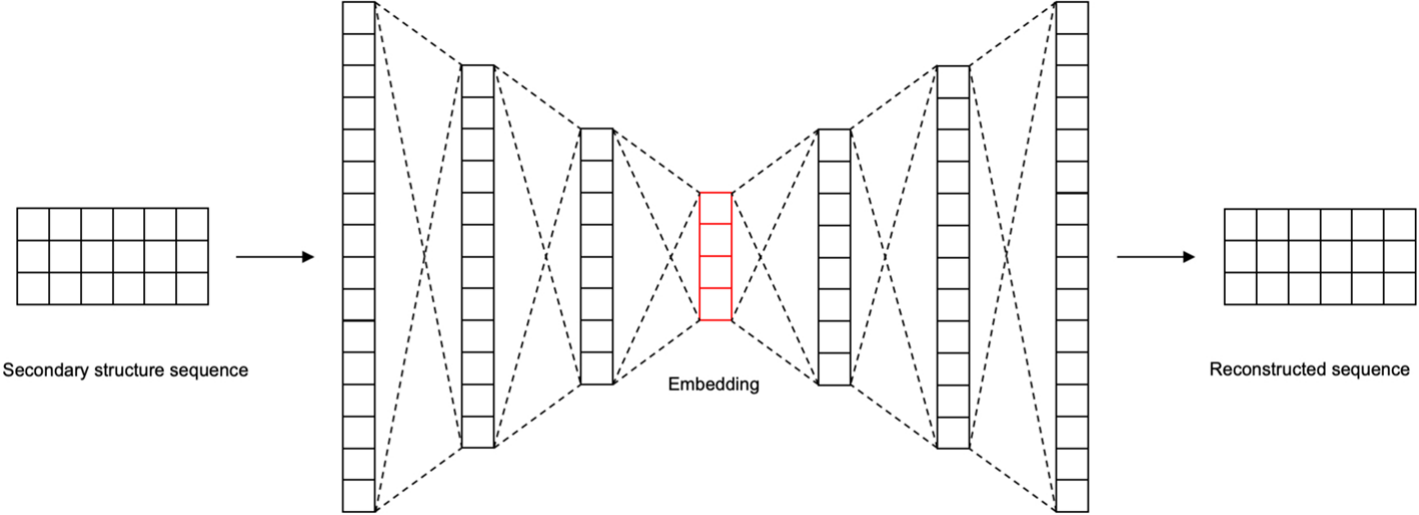
We employed a fully connected autoencoder to derive pre-miRNA secondary structure embeddings. The autoencoder’s input consists of one-hot encoded dot-bracket representations of secondary structures, all of which are standardized to a fixed length of 200. The output of the secondary structure autoencoder is a 64-dimensional vector

We trained the autoencoder to extract embeddings of pre-miRNA secondary structures. The architecture of the autoencoder is visually presented in Fig. 3. As described in the preceding section, the input of the autoencoder was a 4 × 200 tensor. Initially, the input was flattened following which the encoder produces a 64-dimensional embedding vector. Subsequently, the decoder reconstructed the input from this embedding.

We utilized all 36,190 pre-miRNA secondary structures in the training process. We computed the difference for each element in an input sequence compared with its corresponding regenerated sequence using the mean squared error loss function. The training procedure was guided by minimizing the loss value facilitated by the Adam algorithm [42]. To ensure the model’s generalization ability, we conducted training over 10 epochs.

### Architecture of classification model

**Fig. 4:**
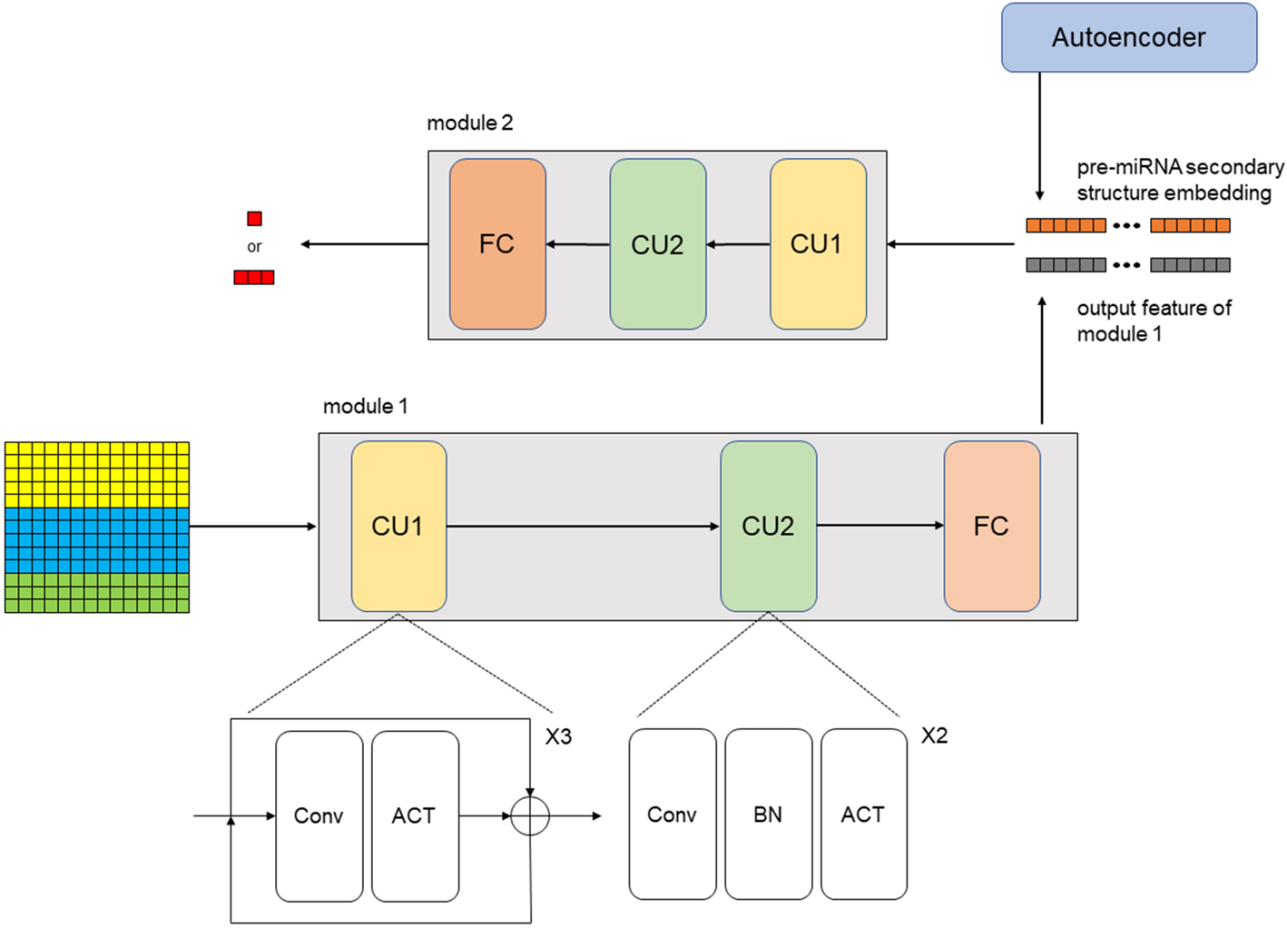
The architecture of DiCleave can be broadly categorized into two modules. The initial module takes a 13-dimensional vector as the input, encompassing both sequential information and secondary structure information of a specific segment. The output of this first module is concatenated with the embedding of the corresponding pre-miRNA secondary structure, which is subsequently given as the input of the second module. The model’s ability to perform binary or multi-class predictions is dependent on its downstream task

The architecture of the classification model was divided into two modules with each module comprising convolution unit 1 (CU1), convolution unit 2 (CU2), and a fully connected unit (FC). Both the binary and multi-class classification models shared the same architecture, differing only in the number of FC neurons and the configuration of the output layer. The input to the proposed model was a 13 × 14 tensor encompassing a nucleic acid sequence, a complementary sequence, and the secondary structure information of a cleavage pattern (Additional file 1: Fig. S1(b)). The architecture of the classification model is illustrated in Fig. 4.

The input tensor representing the cleavage pattern underwent an initial processing step by CU1, which comprises three basic units. Each basic unit of CU1 consisted of a one-dimensional convolutional layer followed by an activation (ACT) layer. To ensure compatibility of the convolution output shape with the input shape, padding was employed. Subsequently, the input tensor was combined with the convolution output through a skip connection. Next, the output of CU1 was further processed by CU2, which comprises two basic units. Each basic unit of CU2 consisted of a convolution layer followed by a batch normalization (BN) layer [43] and an ACT layer. The first and second basic units had 16 and 32 filters in their respective convolutional layers. The extracted features were then passed to an FC layer to reshape the feature structure into a 64-dimensional vector. A dropout operation was implemented within the FC layer to mitigate overfitting during training [44]. The resulting feature vector was then concatenated with the embedding derived from the autoencoder, capturing the pre-miRNA secondary structure. This concatenation produced a 2 × 64 tensor. After that, this tensor underwent further processing through another CU1 within the second module to replicate the identical structure of the first module. In the second module, subtle distinctions emerged in the CU2 and FC layers between the binary and multi-class classification models. The CU2 of the multi-class classification model incorporates an additional convolution layer to expand the input features to a more extensive latent space. Within this convolution layer, the sequential application of a BN layer, a max pooling layer, and an ACT layer (see Fig. 4) was performed. Depending on the specific classification task, the FC layer adjusted the feature tensor to the required shape. In the binary classification model, the output layer consisted of a sigmoid layer, whereas the multi-class model, a softmax layer was employed as the output layer.

The training procedures for the binary classification models on 5’ and 3’ patterns were identical. We randomly allocated 20% of the patterns to form a validation set for monitoring the training process. The remaining 80% of the patterns constituted the training dataset. Owing to the relatively small size of the training dataset, we adopted a modest batch size (20 samples per mini-batch) during training. The binary cross entropy (BCE) was employed as the loss function. To manage the training process, the Adam algorithm was implemented with a learning rate of 0.005 and a weight decay of 0.001. The maximum training epoch was set to 50, and an early stopping mechanism was incorporated to mitigate overfitting. We retained three models that showed the highest accuracy in the validation set.

Although the training process for the multi-class classification model closely resembled that for the binary classification model, some distinctions were apparent. First, we replaced the BCE loss function with a negative log-likelihood loss function. Second, we assigned varying weights to each class when computing the loss value. The number of negative patterns was twice that of the 5’ and 3’ positive patterns, respectively. Consequently, the weight of the negative patterns was set to 0.5 whereas that of the positive patterns remained at 1.

### Ablation models

We used two ablation models to assess the effectiveness of the proposed architecture. In ablation model 1 (AM1), the output from the first module was directly processed by the second module without addition of the pre-miRNA secondary structure embeddings. Conversely, in ablation model 2 (AM2), all CU1 components were removed, signifying that the original input information for each module was not enhanced prior to the convolution process. Both AM1 and AM2 followed the same training procedure as the proposed model with minor adjustments to the layer parameters.

### Performance evaluation

In the context of binary classification, we computed several metrics to assess the model’s performance in the independent test sets. These metrics included accuracy (*ACC*), specificity (*Spe*), sensitivity (*Sen*), F1 score, and Matthews’ correlation coefficient (*MCC*) [45]. The definitions of these binary classification metrics are provided below:

$$Acc=\frac{TP+TN}{TP+TN+FP+FN},$$

$$Spe=\frac{TN}{TN+FP},$$

$$Sen=\frac{TP}{TP+FN},$$

$$F1= \frac{2\times TP}{2\times TP+FP+FN},$$

$$MCC= \frac{TP\times TN-FP\times FN}{\sqrt{\left(TP+FP\right)\times \left(TP+FN\right)\times \left(TN+FP\right)\times (TN+FN)}},$$

where $TP$, $TN$, $FP$, and $FN$ denote the counts of true positives, true negatives, false positives, and false negatives, respectively.

For the evaluation of the multi-class classification models, we adopted a macro-averaging approach for $Acc$, $Spe$, and $Sen$ to calculate the average of each metric across all classes. In addition, we utilized an extension of the binary MCC to the multi-class scenario for evaluation [46]. MCC for the multi-class classification is defined as below:

$${MCC}_{multi}=\frac{c\times s-{\sum }_{k}^{K}{p}_{k}\times {t}_{k}}{\sqrt{\left({s}^{2}-{\sum }_{k}^{K}{p}_{k}^{2}\right)\times ({s}^{2}-{\sum }_{k}^{K}{t}_{k}^{2})}},$$

where ${t}_{k}$ is the number of occurrences for class $k$, ${p}_{k}$ signifies the number of predictions for class $k$, $c$ denotes the total number of samples correctly predicted, and $s$ represents the total number of samples.

## Results

### Performance of binary classification models

**Fig. 5:**
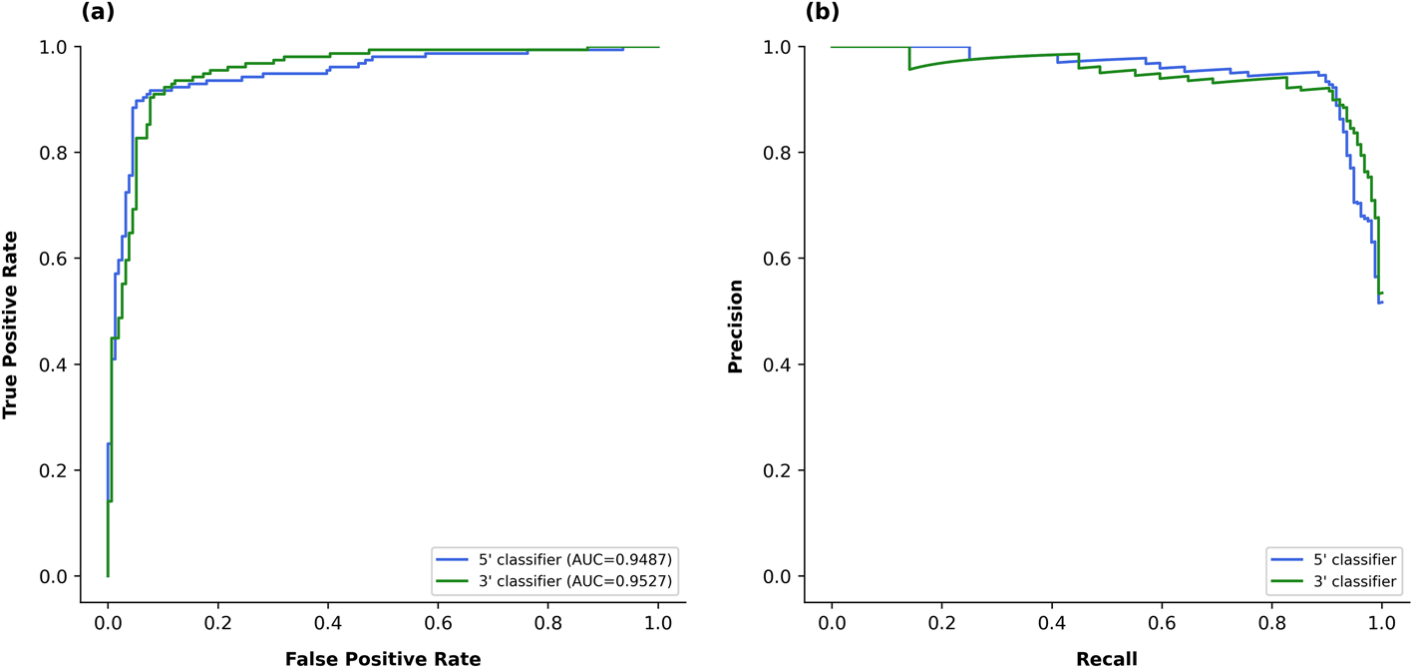
Receiver-operating characteristic curves (**a**) and precision-recall curves (**b**) for DiCleave in the binary classification task. Blue and green lines indicate the performance of the 5’ classifier and the 3’ classifier, respectively

The performance of the best models among replications is listed in Table 2. The test set was balanced, containing an equal number of positive and negative patterns (see Sect. "Methods"). There were no substantial differences between the performances of the models for 5’ and 3’ patterns; both models achieved accuracy, specificity, sensitivity, and F1 scores of 0.90 or higher, whereas MCC reached 0.82. Receiver operating characteristic (ROC) curves and precision-recall (PR) curves for DiCleave in the binary classification task are shown in Fig. 5. Although the area under the ROC curve (AUC) score of the 3’ pattern model was marginally higher than that of the 5’ pattern model, it could be concluded that both models demonstrated nearly identical performance. On the other hand, the performance on an unbalanced test set, which was created by randomly selecting 50 positive patterns and all negative patterns from the original test set, showed a slight decrease compared with that on the balanced dataset, although the F1 score and MCC remained above 0.80 and 0.75, respectively (Additional file 1**: **Tables S1 and S2).

**Table 2:** Performance of different models

		Accuracy	Specificity	Sensitivity	F1 Score	MCC
5’ prediction	DiCleave	**0.9135**	0.9103	**0.9167**	**0.9137**	**0.8269**
	AM1	0.8782	**0.9167**	0.8397	0.8733	0.7587
	AM2	0.8910	0.8782	0.9038	0.8924	0.7823
3’ prediction	DiCleave	**0.9103**	0.9167	**0.9038**	**0.9097**	**0.8206**
	AM1	0.8782	**0.9295**	0.8269	0.8716	0.7604
	AM2	0.8942	0.8974	0.8910	0.8939	0.7885
Multi-class prediction	DiCleave	**0.8942**	**0.9402**	**0.8900**	**0.8931**	**0.8302**
	AM1	0.8686	0.9274	0.8686	0.8681	0.7899
	AM2	0.8734	0.9302	0.8868	0.8740	0.8029

AM1: Ablation model without pre-miRNA secondary structure embeddings

AM2: Ablation model without convolution unit 1 (CU1)

The proposed model outperformed the ablation models in all classification tasks (Table 2). AM1, which lacked pre-miRNA secondary structure embeddings, exhibited the lowest accuracy in all classification tasks. Whereas AM1 achieved the highest specificities in the binary classification task, its sensitivities were the lowest. Conversely, the inclusion of secondary structure embeddings notably enhanced the overall performance of AM2, resulting in significantly improved sensitivities at the acceptable cost of decreased specificity. However, the performance of AM2 was lagged behind that of DiCleave. This can be attributed to the absence of CU1, which hindered the preservation of information within the input.

**Fig. 6:**
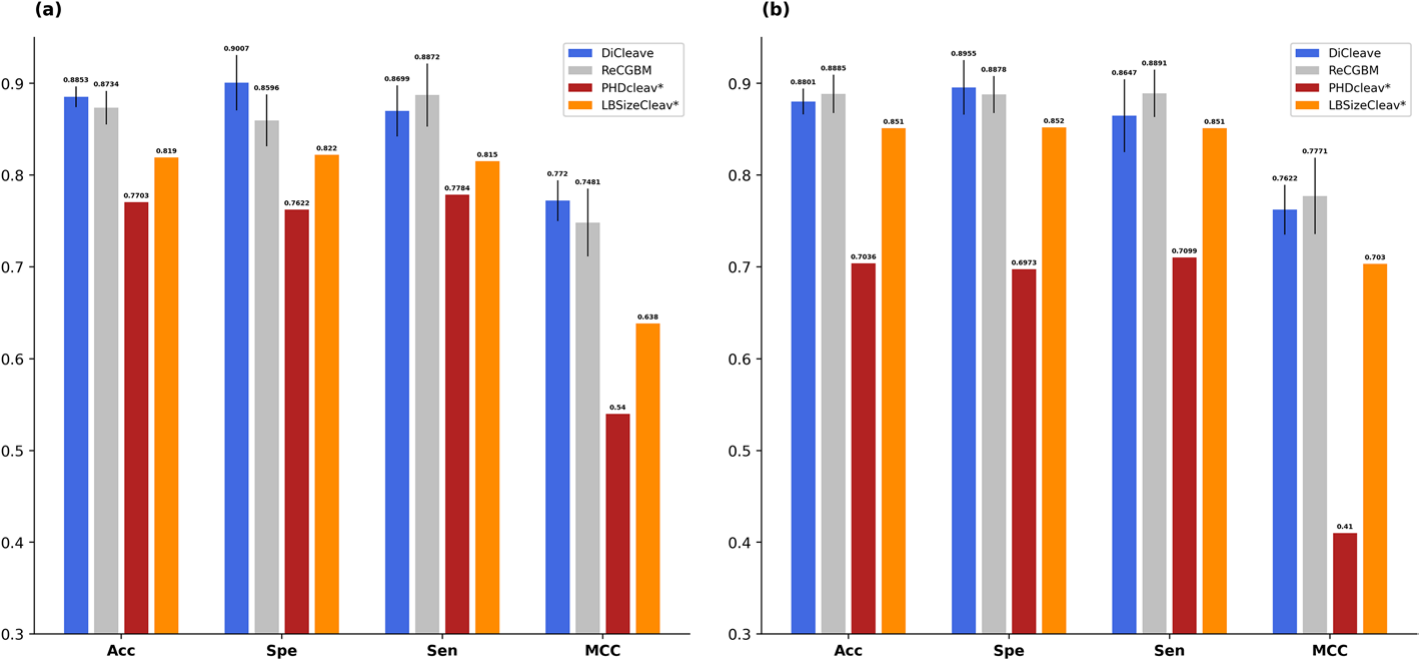
Performance comparison between DiCleave and other existing methods for binary classification tasks for both **a** the 5’ cleavage site classification task and **b** the 3’ cleavage site classification task. DiCleave and ReCGBM show average performances of 10 replications. The performances of PHDcleav and LBSizeCleav (indicated by an asterisk) are sourced from their original articles [28, 29]

To assess the effectiveness of our proposed models, we compared them with ReCGBM. We computed the average performance of DiCleave and ReCGBM across 10 replications with different initial conditions to provide a comprehensive view of their performance. As shown in Fig. 6 and Additional file 1: Tables S3 and S4, the performance of our models surpassed the average performance of ReCGBM in 5’ pattern prediction. In addition, our models demonstrated similar performance in 5’ and 3’ pattern predictions whereas ReCGBM exhibited unequal performance between 5’ and 3’ pattern predictions (i.e., inferior performance in 5’ pattern prediction compared with the 3’ pattern prediction). Figure 6 also includes a performance comparison with PHDcleav [28] and LBSizeCleav [29]. The PHDcleav model utilized binary input features and a window size of 14 [28], whereas the LBSizeCleav model employed parameter k = 1 and a window size of 14 [29]. We selected these two models because their input features closely resembled our architecture. As shown in Fig. 6, both SVM-based models struggled to compete with ReCGBM and DiCleave.

### Performance of the multi-class classification model

Our model can perform multi-class classification tasks by adopting the output layer. When presented with a cleavage pattern sequence, our model was able to predict whether the sequence represented a 5’ pattern, a 3’ pattern, or a negative pattern. The performance of the multi-class classification model is summarized in Table 2. It achieved high accuracy, sensitivity, F1 socre of 0.89, and specificity of 0.94. Furthermore, for an unbalanced dataset for the multi-class classification tasks, both the F1 score and MCC exceeded 0.85 and 0.75, respectively (Additional file 1: Tables S1 and S2).

**Fig. 7:**
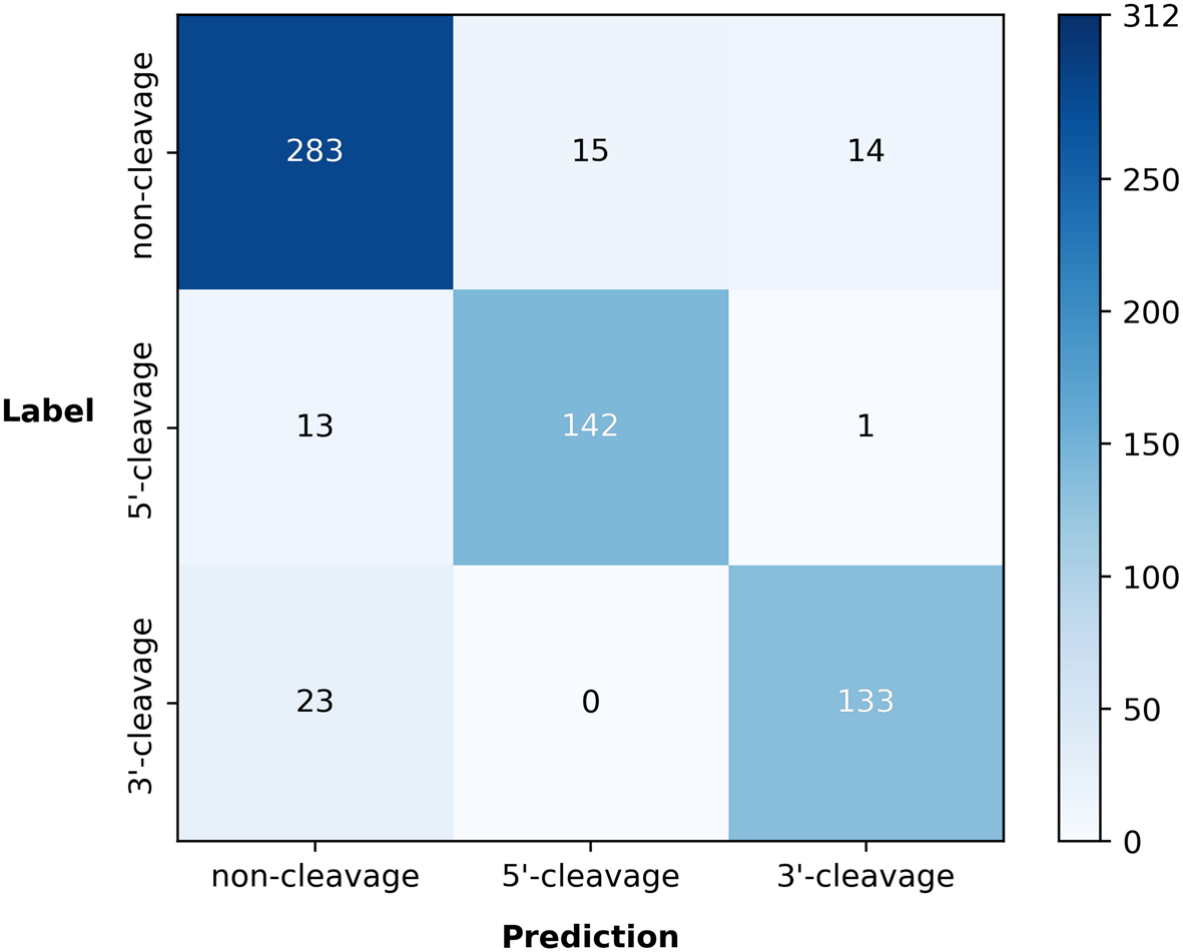
Confusion matrix for the multi-class classification model. Rows and columns represent the counts of true and predicted labels, respectively. The values along the diagonal represent the counts of correctly predicted labels by the model

The confusion matrix for the multi-class classification model is shown in Fig. 7. Only one 5’ pattern sequence was misclassified as a 3’ pattern, and no 3’ patterns were misclassified as 5’ patterns. This outcome highlights our model’s capability to differentiate positive cleavage patterns from different arms. However, there is substantial room for improvement when distinguishing between positive and negative patterns, particularly in the differentiation of 3’ patterns from non-cleavage patterns.

**Fig. 8:**
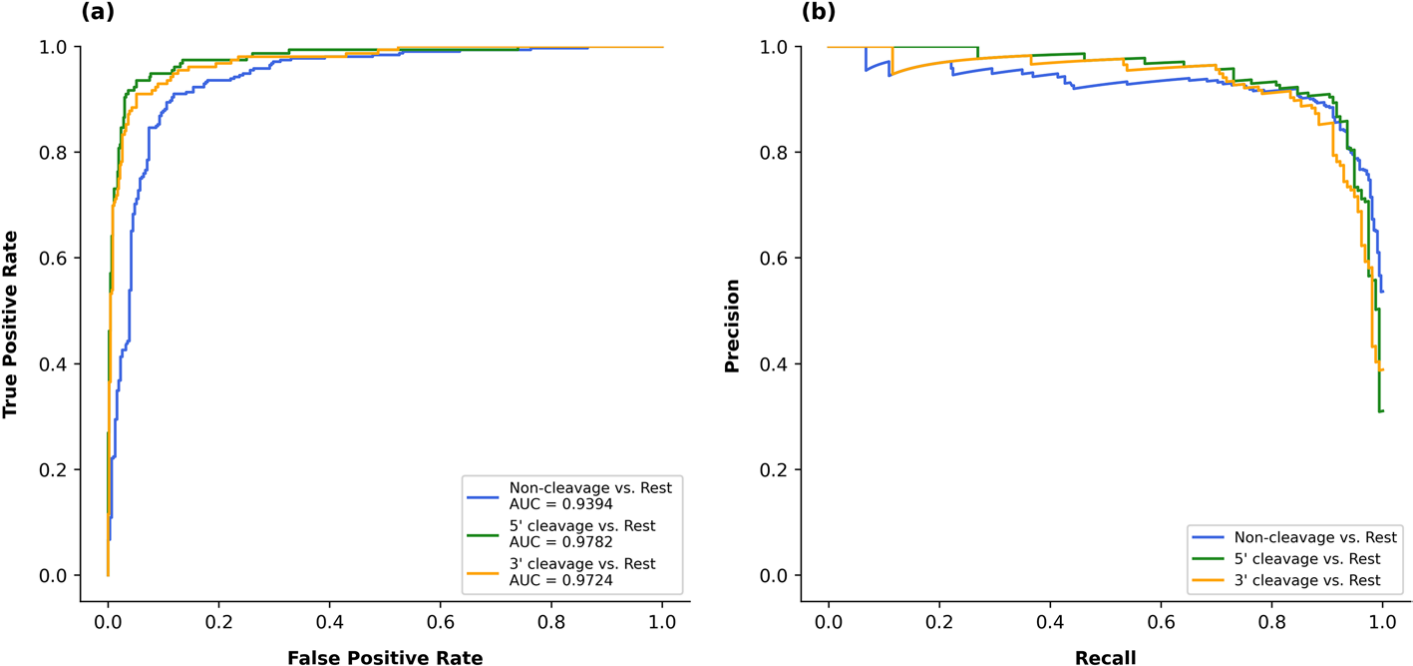
Receiver-operating characteristic curves (**a**) and precision-recall curves (**b**) for the multi-class classification model. In this experiment, the model was transformed into a binary classifier by designating one class as the positive class and the other two classes as the negative classes

The ROC and PR curves for the multi-class classification model using a one-vs-all strategy are shown in Fig. 8. The AUC scores for both 5’ and 3’ patterns reached 0.97, surpassing that of the negative pattern (= 0.93). In the PR curve, the precision for 3’ patterns decreased more rapidly as recall increased, compared with that for 5’ and non-cleavage patterns.

## Discussion

We introduced a DNN model to predict the presence of a human Dicer cleavage site within a short sequence segment, defined as a cleavage pattern. Our model’s input consisted of a combination of one-hot encodings for sequences, including pattern sequences and their complementary sequences, and secondary structure information. Rather than merging these inputs into twelve types of dimers that include combinations of the four bases (“A”, “C”, “G”, “U”) and the three secondary structure indicators (“(”, “.”, “)”) [47], we opted to stack them. This approach was chosen to avoid creating a sparse input space that could yield an unnatural convolution output. The combination of sequence patterns and the secondary structure embeddings extracted by the autoencoder significantly improved the discriminative capacity of the DNN model, resulting in accuracies of 0.91 and 0.89 for the binary and multi-class classification tasks, respectively. This result demonstrated that incorporating pre-miRNA secondary structure embeddings and leveraging the shortcut structure of CU1 significantly enhanced the performance of the model. It is worth noting that the multi-class classification model was trained with an unbalanced dataset in terms of the number of patterns for each class. Although no further measures were taken to address this imbalance apart from adjusting the weight of the negative patterns in the loss calculation, our model still yielded satisfactory results. This success could be attributed to the valuable information provided by the secondary structures, which proved effective in distinguishing between cleavage and non-cleavage patterns.

The computational experiments conducted in this study involved a random selection of training and test datasets from the main dataset. However, even when sequences with 80% or higher similarity were pre-filtered from the main dataset using CD-HIT-EST software [48, 49], DiCleave still demonstrated high performance for predicting cleavage patterns. Specifically, the best model among 10 replications achieved an F1 score of 0.85 or higher for both binary and multi-class classification tasks in the balanced case, and 0.80 or higher in the unbalanced case (Additional file 1: Table S5).

One limitation of this study was the relatively small size of the human pre-miRNA dataset used for training the deep learning model, which led to overfitting during training. To mitigate this, we employed a small batch size of 20 samples per mini-batch, as preliminary experiments indicated that a larger batch size caused the models to become stuck in local minima. We set the learning rate to 0.005, which is larger than the default value in PyTorch’s Adam implementation. Low learning rates (e.g., 1e-4 or 5e-4) were found to slow down the training process and hinder model convergence, whereas high learning rates led to overfitting. One potential solution for the small and unbalanced dataset is data augmentation, which can increase the number of training samples. However, effective data augmentation for nucleotide sequences requires expert knowledge in biology. Another limitation pertains to the definition of the cleavage pattern within pre-miRNA sequences. In this study, a cleavage pattern was defined as a 14-nt-long sequence with a Dicer cleavage site at the center. However, in real scenarios, the cleavage site could occur at any position within a given sequence, which could significantly increase the dataset size if each possible position were considered.

Finally, as identified by Liu and colleagues, the features near the pre-miRNA center were observed to have great significance [30], suggesting an intrinsic interplay among the bases within pre-miRNA. Consequently, our future endeavors include the integration of a Transformer-based model, which has the potential to harness these intrinsic features through the attention mechanism [47]. We also aim to create an end-to-end generative model to directly generate mature miRNA sequences from pre-miRNAs based on our cleavage prediction method in forthcoming research.

## Conclusions

We have demonstrated the effectiveness of our deep learning models in predicting the presence of a human Dicer cleavage site within a given pre-miRNA sequence using both its sequence and secondary structure information. Our binary classification model exhibited superior or comparable performance compared with existing models. Furthermore, our model’s ability to function as a multi-class classifier is highly advantageous and practical. This versatility allows our model to make predictions without requiring prior information for any sequence segment, ensuring accessibility to a broad range of data in the miRBase database even when the available information is incomplete.

### Supplementary Information

**Additional file 1.** Supplementary Tables and Figure.

## Data Availability

The original microRNA data were extracted from miRBase available at https://www.mirbase.org. The datasets used in this study, the trained model parameters, and the code implementing our model are publicly available at https://github.com/MGuard0303/DiCleave/.

## Abbreviations

miRNA: MicroRNA
pri-miRNA: Primary microRNA
pre-miRNA: Precursor microRNA
ACC: Accuracy
Spe: Specificity
Sen: Sensitivity
MCC: Matthews correlation coefficient
TP: True positive
TN: True negative
FP: False positive
FN: False negative
