# Supplementary material for: DiCleave: a deep learning model for predicting human Dicer cleavage sites
Source: BMC Bioinformatics. 2024 Jan 9;25:13. doi: 10.1186/s12859-024-05638-4 (PMC10775615; doi:10.1186/s12859-024-05638-4)
Supplement: Supplementary file 1 — Additional file 1. Supplementary Tables and Figure. [file 12859_2024_5638_MOESM1_ESM.pdf]

DiCleave: a deep learning model for predicting human Dicer cleavage sites

Supplementary Material

Table S1 Description of datasets used in the supplementary experiment.

Dataset	Total # Samples	# Positive Samples	# 5' Samples	# 3' Samples	# Negative Samples
dc_5p_ub	206	50	50	0	156
dc_3p_ub	206	50	0	50	156
dc_ub	412	100	54	46	312
s_80_5p_train	1265	666	666	0	599
s_80_5p_test	300	166	166	0	134
s_80_5p_test_ub	184	50	50	0	134
s_80_3p_train	1235	641	0	641	594
s_80_3p_test	300	164	0	164	136
s_80_3p_test_ub	186	50	0	50	136
s_80_train	2650	1401	719	682	1249
s_80_test	450	236	113	123	214
s_80_test_ub	314	100	53	47	214

dc_5p_ub: unbalanced test set derived from the original 5' model test set; dc_3p_ub: unbalanced test set derived from the original 3' model test set; dc_ub: unbalanced test set derived from the original multi-classification model test set; s_80_5p_train: training set with 80% threshold of 5' model; s_80_5p_test: test set with 80% threshold of 5' model; s_80_5p_test_ub: unbalanced test set with 80% threshold of 5' model; s_80_3p_train: training set with 80% threshold of 3' model; s_80_3p_test: test set with 80% threshold of 3' model; s_80_3p_test_ub: unbalanced test set with 80% threshold of 3' model; s_80_train: training set with 80% threshold of multi-classification model; s_80_test: test set with 80% threshold of multi-classification model; s_80_test_ub: unbalanced test set with 80% threshold of multi-classification model.

Table S2 Performance of the best DiCleave model on unbalanced test sets.

Model	Accuracy	Specificity	Sensitivity	F1 Score	MCC
dc_5p_ub	0.9029	0.9103	0.8800	0.8148	0.7533
dc_3p_ub	0.9223	0.9167	0.9400	0.8545	0.8084
dc_ub	0.9029	0.9366	0.8936	0.8541	0.7755

Table S3 Performance of DiCleave model for 10 replications.

DiCleave		Accuracy	Specificity	Sensitivity	F1 Score	MCC
5' prediction	Best	0.9135	0.9432	0.9103	0.9126	0.8271
	Avg.	0.8853	0.9007	0.8699	0.8834	0.7720
	S.D.	0.0112	0.0254	0.0278	0.0302	0.0278
3' prediction	Best	0.9103	0.9487	0.9103	0.9103	0.8205
	Avg.	0.8801	0.8955	0.8647	0.8799	0.7622
	S.D.	0.0141	0.0251	0.0397	0.0297	0.0397
Multi-class prediction	Best	0.8910	0.9412	0.8953	0.8910	0.8270
	Avg.	0.8628	0.9235	0.8610	0.8618	0.7811
	S.D.	0.0151	0.0091	0.0178	0.0152	0.0242

Table S4 Performance of ReCGBM model for 10 replications.

ReCGBM		Accuracy	Specificity	Sensitivity	MCC
5' prediction	Best	0.9006	0.9038	0.9295	0.8021
	Avg.	0.8734	0.8596	0.8872	0.7481
	S.D.	0.0184	0.0282	0.0344	0.0370
3' prediction	Best	0.9295	0.9295	0.9295	0.8590
	Avg.	0.8885	0.8878	0.8891	0.7771
	S.D.	0.0208	0.0201	0.0258	0.0416

Note that ReCGBM neither reports F1 score and nor supports multi-class classification.

Table S5 Averaged performance of models trained from CD-HIT-EST-processed datasets.

Model		Accuracy	Specificity	Sensitivity	F1 Score	MCC
s_80_5p	Best	0.8933	0.9552	0.8434	0.8974	0.7943
	Avg.	0.8603	0.8666	0.8554	0.8713	0.7227
	S.D.	0.0154	0.0610	0.0440	0.0146	0.0308
s_80_5p_ub	Best	0.9293	0.9552	0.8600	0.8687	0.8204
	Avg.	0.8707	0.8664	0.8820	0.7908	0.7112
	S.D.	0.0360	0.0610	0.0374	0.0404	0.0549
s_80_3p	Best	0.8667	0.8529	0.8780	0.8780	0.7310
	Avg.	0.8377	0.8206	0.8519	0.8514	0.6736
	S.D.	0.0122	0.0322	0.0306	0.0127	0.0239
s_80_3p_ub	Best	0.8817	0.8603	0.9400	0.8103	0.7416
	Avg.	0.8489	0.8206	0.9260	0.7679	0.6835
	S.D.	0.0197	0.0322	0.0297	0.0223	0.0298
s_80	Best	0.8756	0.9344	0.8846	0.8779	0.8067
	Avg.	0.8500	0.9205	0.8589	0.8523	0.7690
	S.D.	0.0175	0.0098	0.0194	0.0176	0.0255
s_80_ub	Best	0.8822	0.9139	0.8404	0.8417	0.7567
	Avg.	0.8363	0.9081	0.8504	0.8048	0.7011
	S.D.	0.0281	0.0109	0.0200	0.0253	0.0364

Avg. denotes the averaged performance for 10 independent models. S.D. denotes standard deviation.

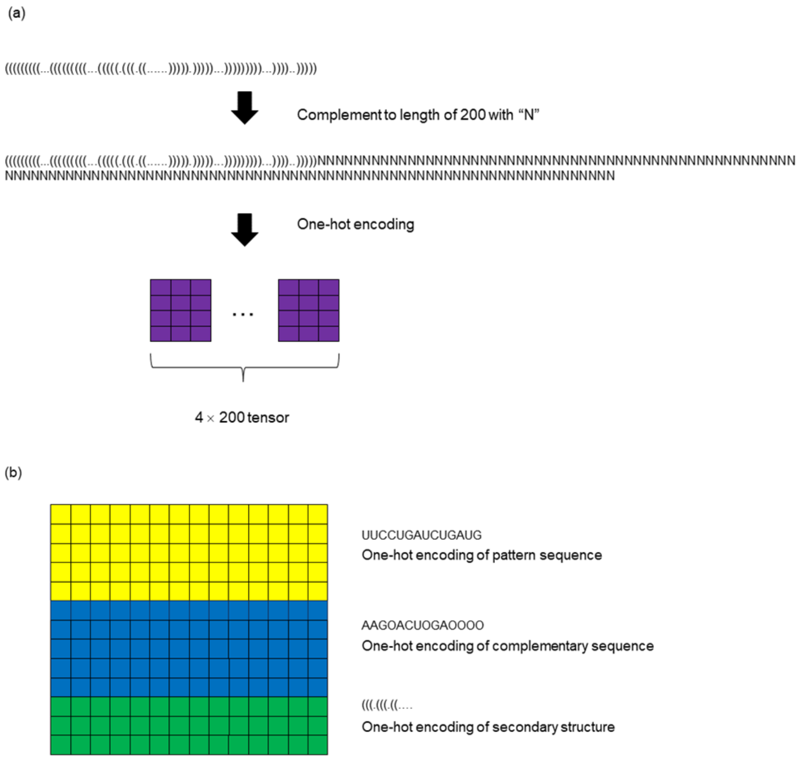

Fig. S1 (**a**) One-hot encoded full-length secondary structure as the input of the autoencoder. Any sequence shorter than 200 nt is complemented with the placeholder "N". (**b**) One-hot encoded DiCleave input. The yellow part is the encoding of the cleavage pattern sequence. Note that token "O" is redundant. The blue part and the green part are the encoding of the complementary sequence and the secondary structure of the pattern, respectively. All three parts are stacked to form a 13 × 14 tensor.
